# Congenital Symptomatic Scaphoid-Trapezium Coalition in a Pediatric Patient With VACTERL Association

**DOI:** 10.1016/j.jhsg.2026.100961

**Published:** 2026-02-28

**Authors:** Rushil Gupta, Atharva M. Bhagwat, Rohun Gupta, Louisa B. Ragsdale, Brian A. Mailey

**Affiliations:** ∗ Saint Louis University School of Medicine, Saint Louis, MO; † Division of Plastic and Reconstructive Surgery, Department of Surgery, Saint Louis University School of Medicine, Saint Louis, MO

**Keywords:** Carpal coalition, Congenital deformity, Pediatric

## Abstract

Carpal coalition is the union between two or more carpals that should otherwise have uninterrupted mobility. Of those reported among pediatric populations, the vast majority are lunotriquetral (69.2%) or capitohamate (17.3%), while scaphotrapezial (ST) is among the most rare. Here, we report a case of a pediatric patient with symptomatic congenital ST coalition as part of the VACTERL association that resolved with surgical intervention. A 9-year-old right-hand-dominant girl with consistent right wrist and snuffbox pain following an incidental fall-on-outstretched hand was later revealed to be symptomatic of congenital ST coalition and unrecognized Blauth grade 1 thumb hypoplasia. Her medical history was notable for a ventricular-septal defect and tracheoesophageal fistula, suggesting a possible VACTERL association. Initial radiographs revealed no acute fractures, but demonstrated developmentally abnormal carpus. The patient experienced continued pain in the radial aspect of her right wrist and first carpometacarpal joint for 5 months despite immobilization and conservative treatment. Physical examination revealed carpometacarpal joint tenderness, unrecognized thumb hypoplasia with underdeveloped thenar musculature, weakened thumb grip, and reduced oppositional movement. Magnetic resonance imaging confirmed hypoplastic thumb and ST coalition, with genetic testing consistent VACTERL association. Surgical management included a right ST-coalition resection, partial carpectomy of the proximal trapezium and distal scaphoid, and fat grafting to the ST interval. The procedure was successful, with no postoperative complications. The patient reported considerably decreased pain with activities that previously elicited pain such as writing and sports. Carpal coalition is a rare congenital abnormality, typically asymptomatic and found incidentally on radiographs. Most cases are often resolved conservatively, though surgery may be indicated for those with continued pain and impairment. For pediatric patients who may have other association/syndrome indications (e.g., VACTERL and Holt-Oram) and otherwise continual wrist pain failing to respond to conservative therapy, carpal coalition may be a contributing factor to their symptoms.

Carpal coalition (CC) refers to the union between two or more carpals that should otherwise allow for uninterrupted joint mobility. Coalitions may be congenital, presenting either as part of a syndrome or in isolation, or acquired following an inflammatory, traumatic, or iatrogenic course.^1^, ^2^, ^3^ Syndromic-associated presentations typically involve bones from more than one row, while those in isolation involve two or more bones within the same row.^1^^,^^2^ Congenital coalitions result from a failure of segmentation during embryological development and typically present as asymptomatic, incidental radiological findings; however, some cases have noted symptomatic presentations secondary to injury or biomechanical stress.^1^^,^^2^^,^^4^

Across adult and pediatric populations, lunate-triquetral coalitions have been noted to overwhelmingly be most frequent, though to varying extents. Previous studies have reported lunate-triquetral coalitions in 89.3% of adult and 69.2% of pediatric presentations, followed by capitate-hamate at 3.9% and 17.3%, respectively.^2^^,^^5^ Among pediatric populations, CCs have been reported to occur at rates of 5 in 1,000, with the highest prevalence among African American populations at 15.6 in 1,000.^5^ These are often associated with syndromes or other congenital malformations, including Holt-Oram, Turner, and Ellis van Creveld syndromes^5^; however, isolated cases of congenital CC have also been reported.^1^, ^2^, ^3^

Previous studies have notably demonstrated scaphoid-trapezium (ST) coalition to be among the rarest forms of CC, consequently resulting in a paucity of literature for the presentation.^1^ In this case report, we document the case of a pediatric patient with unilateral ST coalition and hypoplasia of her thenar eminence and thumb, noted following conservative management of a distal radius fracture.

## Case Report

**Figure 1:**
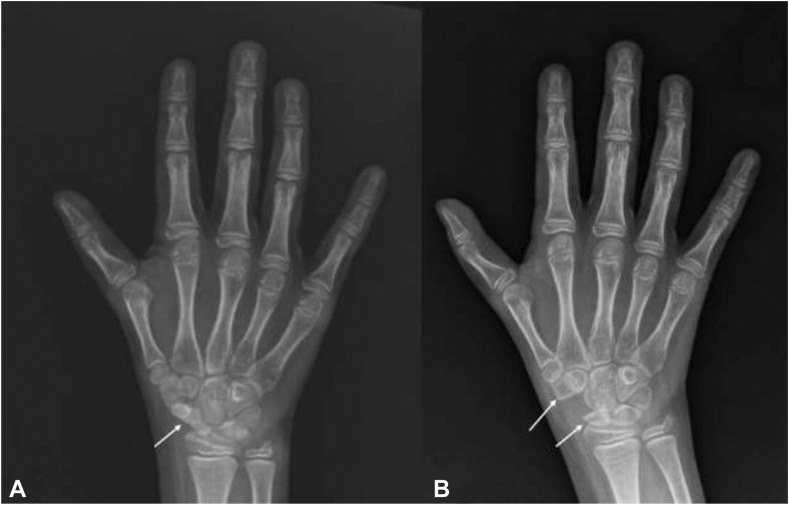
**A** Baseline preoperative radiograph of the right hand with an arrow demonstrating the region of flattening and sclerotic appearance of the scaphoid with interface irregularity at the trapezium, indicative of nonosseous coalition. **B** Six-month postoperative radiograph of the right-hand wrist depicting scaphotrapezial coalition resection along with partial carpectomy of the proximal trapezium and distal scaphoid (arrows).

A 9-year-old right-hand-dominant girl presented to an outside hospital emergency department following a fall on an outstretched hand. Imaging demonstrated no acute fracture but with snuffbox tenderness being present, the patient was placed in a cast and referred to hand surgery for further evaluation. The patient was seen 3 weeks after the date of injury by a hand surgeon, who conducted repeat plain films, which demonstrated no acute fracture but did raise concern for a developmentally abnormal carpus (Fig. 1). Given these concerns, the patient was referred to hand surgery, where she was noted to have thenar wasting, weak pinch grip of her thumb, and no pain to her snuffbox. In addition, her medical history included a ventricular-septal defect and tracheoesophageal fistula, suggesting a high likelihood of VACTERL association with differentials, including Holt-Oram syndrome. Given the patient’s examination with hypoplastic thenar musculature, clinical history, and imaging, she was referred to hand therapy for thenar strengthening with plans for follow-up in 2 months.

**Figure 2:**
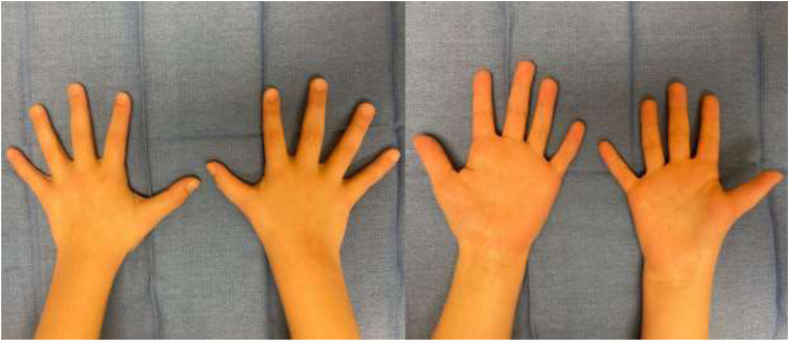
Preoperative volar and dorsal images depicting right thumb hypoplasia along with atrophy of the thenar musculature without notable atrophy of the hypothenar musculature.

At her 2-month follow-up, the patient was noted to have difficulty with fine hand motions, including buttoning without any loss of strength and pain. Because of the timeline of minimal casting, it was hypothesized that the patient’s thenar atrophy was congenital rather than secondary to denervation from compression, and the plan was to continue with therapy. The patient was followed up 5 months from her last clinic visit with concerns for pain along the radial aspect of her right wrist and first carpometacarpal joint. She reported inability to play volleyball and difficulty playing sports, writing, and overall use of her right hand. Physical examination revealed moderate tenderness to the carpometacarpal joint space, severe thenar wasting without hypothenar wasting, weak pinch grip of the right thumb compared to left, and intact yet notable weakness of thumb opposition, adduction, abduction, flexion, and extension (Fig. 2). EMG demonstrated no acute findings. MRI was conducted which revealed a hypoplastic thumb (Blauth grade 1), along with fibrous ST coalition. Surgical and conservative options were discussed, and the family elevated to proceed with operative intervention.

The patient underwent right scaphotrapezial coalition resection, partial carpectomy of the proximal trapezium and distal scaphoid, and fat grafting to the scaphotrapezial interval. A standard Wagner incision was used to gain access to the carpal space overlying the ST coalition. An osteotomy was performed at the proximal resection, and the segment of cartilage connecting the scaphoid and trapezium was removed with a rongeur. Given that the trapezium appeared to be elongated on imaging and direct visualization, an osteotomy was performed on the enlarged portion of the trapezium and removed with a rongeur. A segment of fat was then harvested from the ulnar volar forearm and placed between the scaphoid and trapezium interval (Figs. 1 and 2). The surgical sites were then closed, and the patient was placed in a thumb spica cast with plans for a total of 6 weeks of immobilization.

The patient’s postoperative course was uncomplicated with no signs of infection or pain. At 6 weeks, her cast was removed and she was instructed to begin hand therapy. The patient reported moderate pain localized to the surgical site, but limited pain with activities that previously elicited pain, such as writing. The patient was re-evaluated 9 months after surgery and noted to have complete resolution of her pain and prior symptoms along with thumb hypoplasia with excellent compensation. Following surgical intervention, the patient was able to write and play sports, both of which were activities that she was unable to perform prior to surgery.

## Discussion

The present case illustrates an unusual finding of a patient presenting with a symptomatic, unilateral ST coalition. This type of coalition is relatively uncommon, as most reported CCs involve the lunate and triquetrum.^6^^,^^7^ The coalition was identified incidentally on imaging performed following concerns for a wrist fracture due to a fall-on-outstretched hand. Carpal coalitions are rare congenital abnormalities, typically discovered incidentally on imaging because of unrelated reasons such as trauma or wrist pain. A study by Ogut et al^8^ found that conventional imaging techniques, including plain films and computed tomography, were highly accurate in diagnosing carpal coalitions.

The coalitions are the result of failed segmentation of carpal cartilaginous precursors during embryonic development.^1^^,^^2^ Under normal conditions, the carpals develop from separate cartilaginous centers and differentiate into distinct bones. However, when this process is disrupted, a coalition can form between adjacent carpals.

Treatment of carpal coalition varies based on patient symptoms. Conservative management is recommended for patients who are grossly asymptomatic, with treatment revolving around frequent monitoring and serial examinations. For patients who experience mild wrist pain, lifestyle changes and a multimodal approach, including rest, immobilization, nonsteroidal anti-inflammatory drugs, and physiotherapy, are suggested.^9^ Surgical treatment options for patients who experience severe pain and functional impairment include resection with interposition grafting and proximal row carpectomy.^9^ Resection with autologous fat grafting was the preferred option for this patient as the harvested adipose stem cells would separate the trapezium and scaphoid bone to prevent re-fusion. Additionally, fat grafting is beneficial for maintaining joint mobility as compared to proximal row carpectomy.^10^^,^^11^

There have been several classification systems that have been developed to better describe carpal coalitions and guide surgical management. The first carpal coalition was described by Devilliers Minnaar^12^ in 1952, with four subtypes being described and limitations including the lack of variability seen with nonosseous coalitions.^13^^,^^14^ Burnett^14^ attempted to resolve some of the shortcomings of the Devilliers Minnaar classification by describing coalitions as osseous and nonosseous. Furthermore, despite the introduction of several classification systems, there have been none that have documented or included associated syndromes.^14^

Previous case reports have also described carpal coalitions to be associated with syndromes such as fetal alcohol syndrome, arthrogryposis multiplex congenital, Turner syndrome, oto-palatodigital syndrome, and Holt-Oram syndrome.^1^, ^2^, ^3^ This patient's medical history included ventricular-septal defect, tracheoesophageal fistula, and thenar hypoplasia. These findings were concerning for VACTERL syndrome, classified as a group of birth defects in which the patient presents with vertebral anomalies, anal atresia, cardiac malformations, tracheoesophageal fistula, renal anomalies, and limb abnormalities. Genetic chromosomal microarray testing confirmed the diagnosis of VACTERL association, suggesting that ST coalition may represent an additional symptom.

There have been several management options for ST coalition and bipartite scaphoids. A study by Stewart and McCombe^15^ found that arthrodesis led to symptomatic relief. Similarly, an additional study by Wharton and Ahearne^16^ demonstrated arthrodesis to be effective in the management of pain secondary to ST coalition. Unlike prior studies, a partial carpectomy was conducted of the trapezium and scaphoid, along with fat grafting, with optimal review, which has not been addressed in prior literature.

Patients may benefit from considering treating CC as a differential for patients with previous congenital abnormalities additionally experiencing wrist/hand pain. Additionally, it is integral that management must be individualized based on coalition type, symptom severity, associated anomalies, and functional impairment. Differential diagnosis may include accessory ossicles, post-traumatic changes, or postinfectious. Limitations of this case study include a follow-up time of <1 year, which may limit generalizability of this study because of the impact of growth not being addressed. In addition, formal objective/functional outcome measures were absent, which restricts the reliability of our postoperative outcomes. Finally, risks including recurrent coalition, joint instability, and altered carpal biomechanics were not specifically assessed. Written informed consent was obtained from the patient for publication of this case report and accompanying images.

## Conflicts of Interest

No benefits in any form have been received or will be received related directly to this article.
